# The use of an imageless robotic system in revision of unicompartmental knee arthroplasty

**DOI:** 10.1002/ksa.12574

**Published:** 2024-12-30

**Authors:** Luca Andriollo, Francesco Benazzo, Virgina Cinelli, Rudy Sangaletti, Calogero Velluto, Stefano Marco Paolo Rossi

**Affiliations:** ^1^ Sezione di Chirurgia Protesica ad Indirizzo Robotico, Unità di Traumatologia Dello Sport, UOC Ortopedia e Traumatologia Brescia Italy; ^2^ Università Cattolica del Sacro Cuore Roma Italy; ^3^ Artificial Intelligence Center Alma Mater Europaea University Vienna Austria; ^4^ IUSS Istituto Universitario di Studi Superiori Pavia Italy; ^5^ Department of Life Science, Health, and Health Professions Università degli Studi Link, Link Campus University Roma Italy

**Keywords:** imageless, knee arthroplasty, revision, robotic, TKA, UKA

## Abstract

**Purpose:**

The application of robotics in revision arthroplasty particularly from unicompartmental knee arthroplasty (UKA) to total knee arthroplasty (TKA), is underexplored. The purpose of this study is to describe the surgical technique of an imageless robotic system used in the revision of UKA to TKA and to evaluate short‐ to mid‐term outcomes.

**Methods:**

This prospective study includes 35 patients treated from May 2020 to July 2023. Demographic data of the patients were gathered and the reasons for needing revision surgery were assessed. All patients were clinically evaluated preoperatively and at the final follow‐up of 31.3 ± 12.1 months, using the Western Ontario and McMaster Universities Arthritis Index (WOMAC), Oxford Knee Score (OKS), Forgotten Joint Score (FJS‐12), Numerical Rating Scale (NRS) and range of motion (ROM). Additionally, a radiographic evaluation was performed, and implant survival was assessed by analyzing complications at final follow‐up.

**Results:**

In 88.6% of the patients, a primary Posterior Stabilized (PS) or Constrained Posterior Stabilized prosthetic implant was used, with 11.4% of patients requiring a varus‐valgus constraint implant. In 71.4% of the cases, a thinnest size liner of 10 mm was used. The use of the robotic system was never aborted for any reason. At final follow‐up, the implant survival rate was 97.14%. Average OKS increased from 31.4 ± 9.4 to 41.5 ± 4.3, FJS‐12 from 47.3 ± 19.3 to 80.7 ± 8.9; WOMAC at final follow‐up was 17.8 ± 8.7, from 53.5 ± 21.3 preoperatively. Analyzing ROM, NRS and patient‐reported outcome measures, there were significant differences in each parameter between prerevision surgery and final follow‐up.

**Conclusions:**

This study highlights that in a cohort of patients undergoing robotic‐assisted conversion from UKA to TKA, the use of an imageless procedure incorporating intraoperative bone morphing and alignment based on a functional philosophy has proven to be safe and has yielded excellent clinical and radiographic outcomes.

**Level of Evidence:**

Level II, prospective cohort study.

**Abbreviations**
FJS: Forgotten Joint Score
NRS: Numerical Rating Scale
OKS: Oxford Knee Score
ROM: range of motion
TKA: total knee arthroplasty
UKA: unicompartmental knee arthroplasty
WOMAC: Western Ontario and McMaster Universities Arthritis Index

## INTRODUCTION

In recent years, robotic‐assisted systems have been increasingly integrated into the realm of orthopaedic surgery, promising enhanced accuracy in implant positioning and alignment. Studies have demonstrated that robotic‐assisted total knee arthroplasty (TKA) can improve component alignment, real‐time control of soft tissue balance and potentially lead to better functional outcomes and implant longevity [9, 22, 29, 33]. However, while the benefits of robotic assistance in primary TKA are well‐documented, its application in revision TKA, particularly from unicompartmental knee arthroplasty (UKA) to TKA, remains under‐explored.

UKA offers distinct advantages such as bone conservation, ligament preservation and restoration of normal knee kinematics [30, 32]. Despite these benefits, UKA has been associated with higher revision rates compared to TKA, as evidenced by national prosthesis registries [17, 18]. The technical demands of UKA and the dependency on surgeon experience further complicate its outcomes [19]. As the prevalence of UKA procedures increases, so does the necessity for subsequent UKA to TKA revisions, which are often complicated by issues such as bone loss, poor bone quality and soft tissue contracture [26, 34]. These challenges underscore the need for advanced techniques that can enhance surgical precision and outcomes in revision scenarios.

The ROSA Robotic Knee System (Zimmer Biomet) aids surgeons in multiple aspects of the surgery such as the distal femoral cut, determining the sizing and positioning of the femoral component, guiding the tibial cut and the ligament balance. This robotic system has two options: an image‐based approach or an image‐less method based on intraoperative landmarks acquisition [2].

It is a reliable system associated with minimal complications and offers a reproducible surgical technique. The imageless robot‐assisted system has shown to deliver excellent outcomes for TKA [1, 6, 22].

At the present time, no study have been published on the use of this system for Revision Surgery. Aim of this study is to describe the surgical technique of this imageless robotic system for revising UKAs to TKAs, to evaluate perioperative data, implant survival and short‐ to mid‐term clinical outcomes. The hypothesis is that this system provides an advantage in the revision procedure, allowing for a careful choice of the degree of constraint and giving satisfactory clinical and survival outcomes.

## METHODS

Patients treated using the imageless robotically assisted technique ROSA Knee System (Zimmer Biomet) for the revision of UKA to TKA were prospectively evaluated. The study included patients treated between May 2020 and July 2023, with all procedures performed at a single high‐volume centre specializing in primary and revision arthroplasty surgery.

Inclusion criteria encompassed medial, lateral, patellofemoral or combined UKA revisions. The study included patients treated either due to the failure of the existing implant or due to the progression of osteoarthritis (OA) in other compartments. All patients in this study underwent revision using the imageless robot‐assisted system. The minimum required follow‐up for inclusion was 12 months postrevision surgery.

Exclusion criteria included incomplete preoperative, intraoperative or follow‐up data, a history of periprosthetic joint infection (PJI, 1 case), and a periprosthetic fracture (1 case).

The demographic data of the patients were gathered, including risk factors like diabetes, active smoking and osteoporosis or osteopenia. For all patients, the reasons for revision surgery were assessed, which included osteoarthritic progression, aseptic loosening, painful prosthesis, joint stiffness or other causes.

All patients were evaluated radiographically with weight‐bearing anteroposterior, lateral, Rosenberg, sunrise view and long leg view x‐rays to assess lower limb alignment and remodelling of the distal femur and proximal tibia. When necessary, a CT scan was requested to assess bone stock. When there was a suspicion of avascular osteonecrosis, magnetic resonance imaging was performed. Data on the types of implants used were collected.

From May 2020 to July 2023, 37 patients were treated for UKA revision with TKA implantation using the imageless robotically assisted technique. At the final follow‐up, no deaths or lack of updated contact were recorded. Two patients were not evaluated as they were not eligible based on exclusion criteria. Therefore, 35 patients were analyzed in this prospective evaluation.

Among these patients, 10 were male (24.2%) and 25 were female (75.8%). The average age at the time of surgery was 69.7 ± 7 years. The surgical side was left in 20 cases (57.1%) and right in 15 cases (42.9%).

The revision was performed in 25 cases on medial UKAs (71.4%), in nine cases on lateral UKAs (25.7%) and in one case on a combined lateral UKA and patellofemoral arthroplasty (2.9%). The cause of revision was tricompartmental OA progression in 28 cases (80%) and complications related to the existing implant in seven cases (20%). Table 1 reports the causes of revision and the type of implant revised. Specifically, in the case of the combined implant there was patellar instability, in four cases there was aseptic loosening, and in two cases there was painful hypercorrection on the coronal axis following medial UKA implantation.

**Table 1:** Data on the causes of revision and the type of implant revised.

Indication	Number	%
UKA failure	7	20
Progression of osteoarthritis	28	80
Primary arthroplasty		
Medial UKA	25	71.4
Lateral UKA	9	25.7
Lateral UKA + patellofemoral arthroplasty	1	2.9

Abbreviation: UKA, unicompartmental knee arthroplasty.

The majority of patients were classified as ASA grade 2, accounting for 85.7% of cases, while 14.3% were classified as ASA grade 3. The average BMI of the patients was 25.5 kg/m² (standard deviation [SD] 5.3). Osteoporosis was observed in 17.1% of the patients, and 22.9% were active smokers. Additionally, 14.3% of the patients had diabetes mellitus, and 2.9% were diagnosed with rheumatoid arthritis.

Regarding the type of anaesthesia used, 91.4% of patients underwent spinal anaesthesia, while 8.6% had general anaesthesia.

The evaluation also covered acute complications like postsurgical local haematoma, vascular injury or nerve injury, as well as follow‐up complications including the rates of readmission or reoperation and their respective causes, such as infection, aseptic loosening and thromboembolic events.

All patients received routine venous thromboembolism prophylaxis with low‐molecular‐weight heparin for 30 days postsurgery (or alternatively, chronic anticoagulant therapy) and routine perioperative prophylactic antibiotic therapy with cefazolin (except in cases of allergy). Postoperative rehabilitation protocols included immediate full weight‐bearing supported by crutches for the first 30 days. No movement restrictions were imposed, and exercises focused on immediate active flexion and extension.

All patients were clinically evaluated preoperatively and at the final follow‐up using the Western Ontario and McMaster Universities Arthritis Index (WOMAC), Oxford Knee Score (OKS) and Forgotten Joint Score (FJS‐12). Pain was assessed using the Numerical Rating Scale. The range of motion (ROM) was evaluated in terms of maximum flexion and extension deficit. The survival of the implants after revision from UKA to TKA using the imageless robot‐assisted system was evaluated.

X‐ray evaluations of preoperative and postoperative images were carried out based on the criteria published by Ewald and Meneghini [11, 24]. Radiographic measurements were taken using the Picture Archiving and Communication Systems (Carestream Health), with an accuracy of 0.1°. Furthermore, during the final follow‐up, radiographic analysis was performed to check for any signs of prosthesis movement.

All patients gave written, informed consent for the handling of their personal data. The study was performed in accordance with the ethical standards of the 1964 Declaration of Helsinki and with the HIPAA regulation. The study was approved by the local ethics committee with IRB Approval No. NK 5022.

### Surgical technique

The patient is placed in a supine position on the operating table, supported laterally and with an adjustable leg holder for varying degrees of knee flexion. The ROSA Knee System (Zimmer Biomet) was employed, following the surgical technique described by Battailer et al. and Rossi and Benazzo [2, 27]. The goal was to achieve neutral alignment within ±3^o^ to ensure optimal gap balancing.

The surgical approach involves using the previous surgical scar, with possible enlargement to ensure adequate visibility of the entire joint. The procedure includes a medial parapatellar arthrotomy. After the initial approach and arthrotomy and the positioning of the reference trackers, the surgeon conducted the knee state evaluation, beginning with the registration of femoral and tibial landmarks.

A key point to note is that the landmarks at the locations where the unicompartmental implant is present are taken directly on the existing prosthesis, without removing the polyethylene liner.

After mapping the bone morphology, the surgeon assessed the ligament competence using a varus‐valgus stress test at various degrees of flexion (0°, 30°, 45°, 60°, 90°, 120°). With the available data, namely the knee's ROM (in degrees), overall limb alignment (OLA), varus/valgus deformity (in degrees) and ligament laxity (in mm) at different angles, the surgeon used dedicated software for intraoperative planning to determine the optimal thickness and angle of bone resection, aiming to achieve a well‐aligned and balanced TKA.

A discrepancy >3 mm (between 3 and 5 mm) between the medial and lateral compartments at 0° and 30° after the distal femoral and proximal tibial cuts, or medial laxity at 60° or 90° of more than 3 mm, indicated the need for a constrained posterior stabilized (CPS) implant as described by Rosso et al. [31]. Discrepancies >5 mm in extension or more than 3 mm between the flexion and extension spaces indicated the need for a varus‐valgus‐constrained implant. The robotic technique allows for real‐time assessment and adjustment of resection thickness, joint gaps and limb alignment during the surgical procedure.

The first step involves making the proximal tibial cut, aiming to achieve a medial proximal tibial angle (MPTA) between 84° and 92°. The tibial cut should be planned and executed just distal to the tibial component, for which it is important to know the thickness, thus also concurrently removing the tibial component with the maximum possible bone stock preservation.

Following the initial cuts, a reassessment is conducted using a tensioner, specifically the Fuzion System (Zimmer Biomet) and the surgical plan is updated as needed before making the distal femoral cut. The distal femoral cut is made just proximal to the femoral component, with the aim, in this case as well, to preserve as much bone stock as possible. The planning for the distal femoral cut takes into account the boundaries for the lateral distal femoral angle (LDFA), which should be between 84° and 92°. After each bone resection, the surgeon can check and confirm the accuracy of the cuts using a dedicated validation tool.

If the measurements confirmed during surgery differ from the initial plan, these deviations can be recorded at each stage on the plan and appropriate adjustments can be made.

By adopting a personalized alignment approach, it's possible to balance the gaps by modifying the implant targets across all three planes. This gives the surgeon the flexibility to determine and adjust the ideal alignment for each patient, refining bone cuts to reduce the necessity for ligament releases. The goal remains to maintain a Hip‐Knee‐Ankle (HKA) angle of 177°–183°.

In addition to choosing the constraint based on previously described gaps, there may be a need to use an L‐CCK (Condylar Constrained Knee, Zimmer Biomet) implant to address femoral bone loss with augments or to elevate the tibia using half or full augments. Indeed, this type of implant is the available option in Europe that allows for the use of augments together with the ROSA Knee System (Zimmer Biomet) during the study phase.

The ROSA Knee System (Zimmer Biomet) facilitates the balancing of the extension gap, while a specialized ‘rotational tool’ is used to ensure balance between the flexion and extension gaps via the Fuzion System (Zimmer Biomet). These adjustment targets are tailored specifically to the individual patient's knee and the existing gaps.

**Figure 1:**
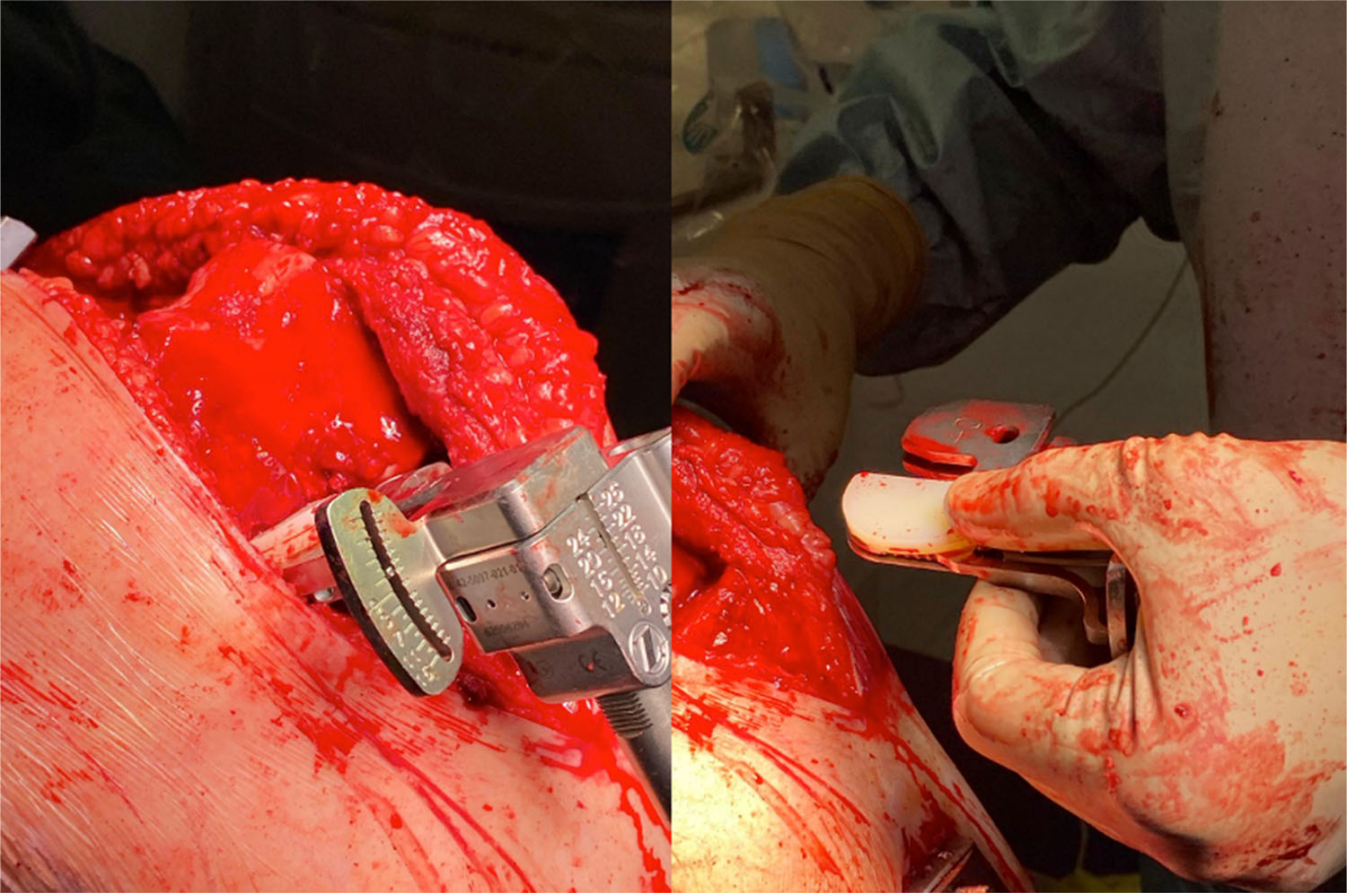
Evaluation of the flexion spaces using the Fuzion System (Zimmer Biomet), with manual application of the liner to mimic the posterior condyle space.

The relevant feature of this technique is that the extension space is evaluated using a dedicated spacer, while the use of the Fuzion System (Zimmer Biomet) in flexion requires a manual adjustment. Indeed, in the evaluation of the rotational alignment, there is a lack of postero‐medial or postero‐lateral condyle due to the removal of the previous implant. At this stage, the liner is used to mimic the posterior condyle to fill the space (Figure 1).

The subsequent step involves positioning the 4‐in‐1 cutting block using the robotic arm based on the evaluation of tension in both flexion and extension. With the trial components inserted, the surgeon can evaluate ligament balance and the degree of any remaining knee deformity. If an imbalance is detected, several options can be considered, such as using a thicker liner, performing additional bone recuts or releasing ligaments. Once the cemented prosthesis is in place, a final assessment can be performed, including any necessary final ligament releases.

The patella is replaced in all patients. At the end of the procedure, after proper placement of the patellar liner, it is also important to assess patellar tracking, which can be further corrected through a lateral release using a pie‐crusting technique on the lateral retinaculum or by adjusting medial tension during capsular suturing.

### Statistical analysis

An independent statistician performed the statistical analysis using SPSS v18.0. Continuous variables were reported as mean and SD, while categorical variables were expressed as frequency distributions and percentages. To determine statistically significant differences, continuous variables between preoperative and follow‐up were compared using the *t* test. The confidence interval was set at 95%, and significance was defined as a *p* < 0.05. Survival analysis was carried out using the Kaplan–Meier method, with revision surgery considered as the failure criterion.

## RESULTS

The average surgical time was 116.1 ± 19.6 min. The average preoperative haemoglobin level was 13.1 ± 0.2 g/L, which dropped to 10.2 ± 0.4 g/L on the first day postoperatively and further decreased to 9.3 ± 0.3 g/L at discharge. Transfusions of packed red blood cells were necessary for three (8.6%) of the patients.

During the revision surgery, a Persona® implant (Zimmer Biomet) with a posterior‐stabilized (PS) liner was used in 26 cases (74.3%), a CPS liner was used in five cases (14.3%) and a L‐CCK implant was used in four cases (11.4%) for varus‐valgus constraint.

**Figure 2:**
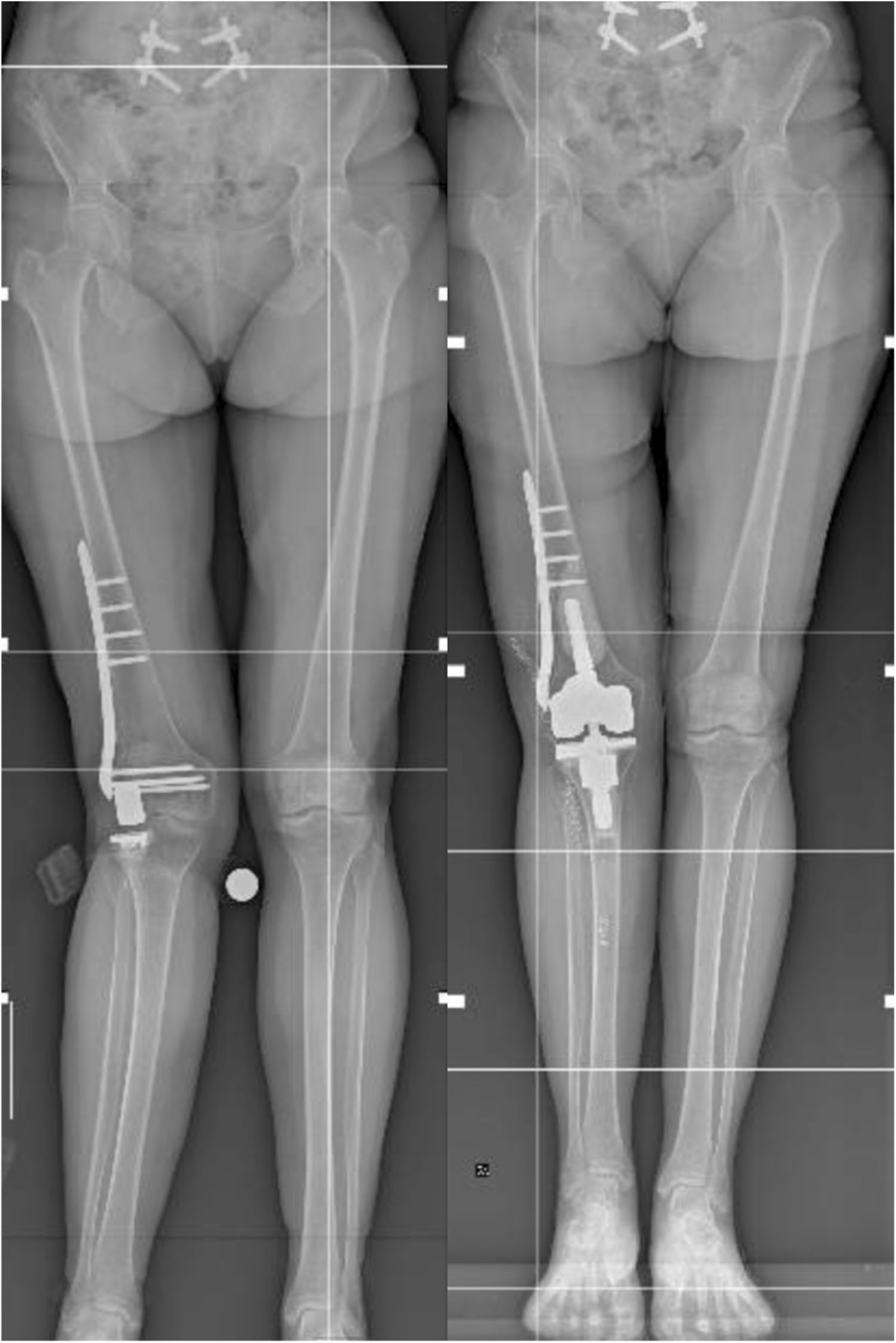
Full‐length standing x‐rays before and after revision in a patient with lateral unicompartmental knee arthroplasty and distal femur fixation, where partial removal of the fixation devices and the use of a tibial cone were also necessary.

In 25 patients (71.4%), the thinnest liner corresponding to 10 mm was used. In five patients (14.3%), an 11 mm liner was used and in another five patients (14.3%), a 12 mm liner was used. In the four patients who required an LCCK implant, femoral augments were used in two cases (5.7%) and tibial augments in two cases (5.7%), consisting of one full tibial block augment of 5 mm and one tibial half block augment of 5 mm. In only one patient, it was necessary to use a tibial cone (Figure 2). The implants used are summarized in Table 2.

**Table 2:** Description of the implants used.

Type of implant	Number	%
Persona PS	26	74.3
Persona CPS	5	14.3
L‐CCK	4	11.4
Liner thickness (mm)		
10	25	71.4
11	5	14.3
12	5	14.3
Implants with augment		
Femoral	2	5.7
Tibial	2	5.7

Abbreviations: CCK, Condylar Constrained Knee; CPS, constrained posterior stabilized; PS, posterior stabilized.

The use of the robotic system was never aborted for any reason. All patients (100%) adhered to the fast‐track rehabilitation protocol. Assisted standing and walking occurred on the day of surgery for 28 (80%) patients, while seven (20%) were assisted on the first postoperative day. The average length of hospitalization was 4.2 ± 1.7 days.

In‐hospital complications were recorded in two (5.7%) cases. In the immediate postoperative period during hospitalization, one patient (2.9%) experienced a complication of intraarticular haematoma, which required an embolization procedure and intraarticular drainage via arthrocentesis. Another patient showed significantly elevated inflammation markers, managed with extended antibiotic therapy and serial monitoring, resulting in an extended hospital stay of up to 11 days postsurgery. The average follow‐up at the final evaluation was 31.3 ± 12.1 months.

Twenty‐five patients (71.4%) were discharged home following the primary TKA rehabilitation protocol [28]. Ten patients (28.6%) were sent to a rehabilitation facility, with an average additional stay of 10.3 ± 3.1 days.

One patient developed joint stiffness with a maximum flexion of 75°. Eleven weeks after the revision surgery, this patient underwent another revision with a decrease in the PS liner from 12 to 10 mm and a proximal transfer of the anterior tibial tuberosity, increasing the patellar height. No other complications such as infection, aseptic loosening or thromboembolic events were reported.

At the final follow‐up, 34 patients were examined, with an implant survival rate of 97.14%.

Clinical Outcomes are summarized in Table 3. The average final OKS was 41.5 ± 4.3. At final follow‐up, 13 patients (38.2%) showed excellent outcomes (OKS > 41), 19 (55.9%) good outcomes (OKS: 34–41) and two (5.9%) fair outcomes. No patient reported a poor (OKS < 27) outcome. The average FJS‐12 at final follow‐up was 80.7 ± 8.9. The average WOMAC score at final follow‐up was 17.8 ± 8.7.

**Table 3:** Comparison of range‐of‐motion, pain and patient‐reported outcome measures between prerevision surgery and final follow‐up.

	Prerevision surgery	Final follow‐up	*p* Value
Maximum of flexion	105.3° (SD 21.4)	119.4° (SD 13.4)	<0.001
Deficit of extension	7.5° (SD 3.5)	0.9° (SD 1.1)	<0.001
WOMAC	53.5 (SD 21.3)	17.8 (SD 8.7)	<0.001
FJS‐12	47.3 (SD 19.3)	80.7 (SD 8.9)	<0.001
OKS	31.4 (SD 9.4)	41.5 (SD 4.3)	<0.001
Pain (NRS 0–100)	57.3 (SD 21.3)	9.1 (SD 3.4)	<0.001

Abbreviations: FJS, Forgotten Joint Score; NRS, Numerical Rating Scale; OKS, Oxford Knee Score; WOMAC, Western Ontario and McMaster Universities Arthritis Index.

**Figure 3:**
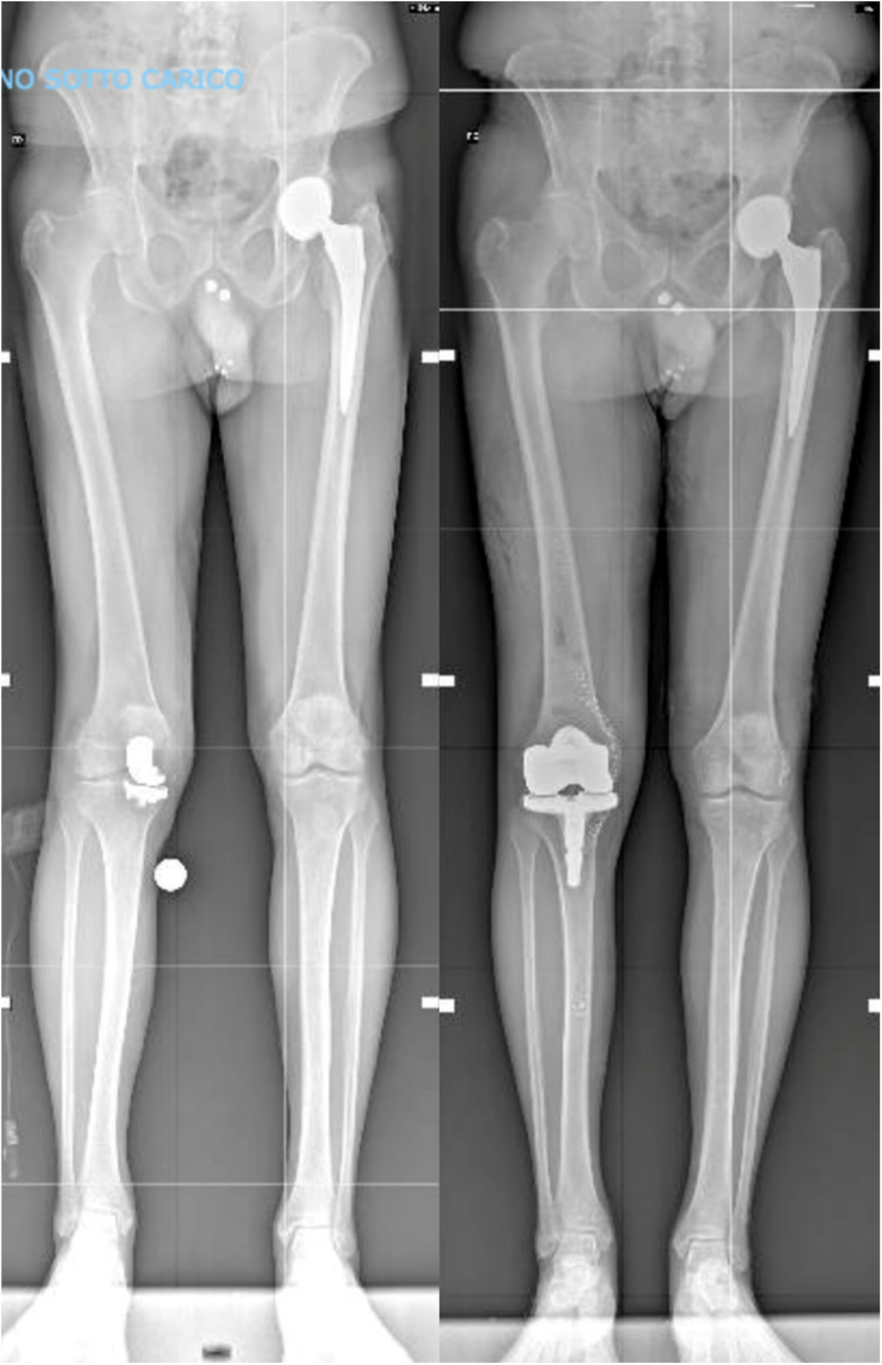
Full‐length standing x‐rays before and after revision in a patient with medial unicompartmental knee arthroplasty, with tricompartmental osteoarthritic progression.

**Figure 4:**
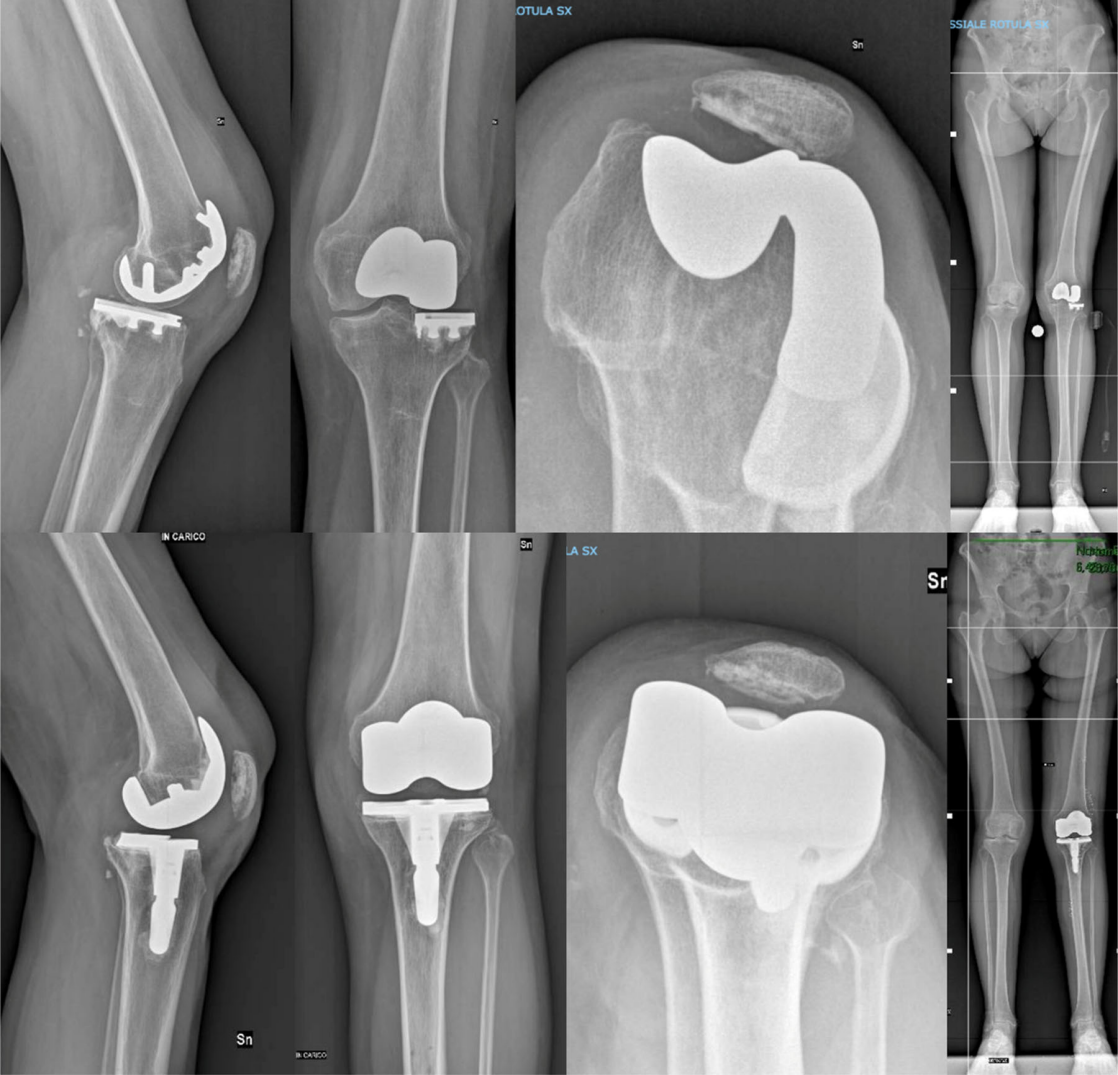
Preoperative and final follow‐up radiographic evaluation of a patient with a lateral unicompartmental and patellofemoral implants, revised using the Rosa Knee System (Zimmer Biomet).

Radiological evaluation showed no significant progressive radiolucent lines. Specifically, one patient with a CCK implant showed minor ( <2 mm) and nonprogressive radiolucent lines around the femur (zone 4). Additionally, three patients (two in the PS group, one in the CPS group) exhibited minor, nonprogressive radiolucent lines beneath the tibial component (zones 1, 3, 6 and 7). Notably, in two cases, these radiolucent lines were already present in the immediate postoperative x‐rays, likely due to the cementation technique used. Implant positioning was evaluated using the angles described by Ewald. All implants were accurately positioned (see Table 4), with an average HKA angle of 179.3° ± 2.3° (Figure 3). There was no evidence of implant loosening in the anterior‐posterior and lateral views or in long‐standing x‐ray (Figure 4).

**Table 4:** Average angles calculated according to the Knee Society total knee arthroplasty roentgenographic evaluation and scoring system.

	Alfa	Beta	Gamma	Delta	HKA
Average angle	91.5	89.1	3.3	88.8	179.3
SD	2.7	1.3	2.1	2.1	2.3

Abbreviations: HKA, Hip‐Knee‐Ankle; SD, standard deviation.

## DISCUSSION

The most important finding of the current study is that imageless robotic conversion of UKA to TKA reached relevant clinical outcomes associated with satisfactory short‐ to mid‐term survival. The described technique didn't show significant complications and allowed a reproducible choice of the implant in terms of level of constraint and liner thickness.

In recent years, there has been an increase in the number of UKA procedures, known for being an effective treatment for unicompartmental knee OA [5, 7, 8, 12, 17].

It is important to note that while robot‐assisted primary procedures have shown success, robot‐assisted revision surgeries have not yet received approval for widespread use [3, 4, 10, 13, 23, 25].

There are only a few studies in the literature on revisions from UKA to TKA performed with the assistance of robotic systems [37]. This study provides a detailed description of a surgical technique performed with an imageless robot‐assisted system.

In a study published by Magruder et al. involving 44 patients, 84% involved medial implants, 11% involved patellofemoral implants and 11% involved lateral implants [20]. In a study published by Mancino et al., 56% of the patients underwent a revision of the medial implant, 25% of a lateral implant and 19% of a patellofemoral arthroplasty [21]. In the study by Tuecking et al., involving 20 patients, all implants were medial [35]. It is worth noting that primary medial UKA are more commonly frequent for OA compared to lateral prostheses, potentially contributing to the higher revision rate observed in medial implants [4].

In this research, revisions were primarily driven by the advancement of tricompartmental OA in 28 cases (80%) and issues associated with the current implant in seven cases (20%). In detail, Magruder et al. reported 75% of revisions due to OA progression, 16% due to aseptic loosening, 4.5% due to unspecified pain, 2.3% due to polyethylene wear and 2.3% due to prosthetic joint infection [20]. In a study published by Mancino et al. involving 16 patients treated with robot‐assisted revision from UKA to TKA, the causes of revision are reported as progression of OA in 62% of cases, pain in 13%, instability in 13%, aseptic tibial component loosening in 6% and valgus collapse in 6% [21]. In contrast, Tuecking et al. reported the reasons for UKAs revision as 40% due to aseptic loosening, 25% due to secondary instability, 15% due to valgus overstuffing and only 20% due to OA progression [35]. However, the small sample size should not be considered representative of the reasons for revision of UKAs, as the existing literature reports that the main cause of revision is the progression of OA in the other compartments [16, 19, 38].

The studies mentioned in the literature have highlighted the benefits of using robotic systems for converting UKA to TKA, emphasizing their advantages. Specifically, they mention improved bone stock conservation, precise and accurate intraoperative bone cuts and minimizing the depth of resection, resulting in less use of stems and augments.

Kalavrytinos et al. reported the initial instance of utilizing robot‐assisted technology to convert a failed UKA to a TKA [14]. The procedure was carried out to address a malpositioned UKAs with a varus deformity, using the Mako system (Stryker) guided by CT scans.

Wallace utilized the same robot‐assisted system to convert four unsuccessful UKAs into TKAs [36]. His findings underscored that robotic‐assisted technology plays a crucial role in conserving bone stock during such conversions. This preservation is facilitated by the fact that preoperative planning does not incorporate the UKA implant into calculations, allowing for precise and accurate intraoperative bone cuts. This approach helps maintain bone integrity and ensures optimal preparation for the TKA procedure.

Yun et al., using a CT‐based robotic system, showed that robot‐assisted revisions result in reduced bone resection compared to traditional methods [38]. Additionally, they noted variations in the utilization of stems and augments; none of the knees in the robot‐assisted group required augments, in contrast to 29% in the conventional group. The authors suggested that computer mapping of the residual bone surface after implant removal was a helpful guide in minimizing resection depth, and the preoperative CT scans were unexpectedly helpful in establishing mechanical alignment and resection depth in the absence of typical anatomic landmarks to guide conventional methods.

Lachance et al. conducted three conversions from patellofemoral prostheses to total knee arthroplasties using the Stryker Mako system (Stryker) based on preoperative CT scans [15]. The revisions were performed due to the progression of OA in the other compartments of the knee.

In contrast, Tuecking et al. employed an imageless robotic system, similar to our research [35]. They highlighted similar alignment outcome parameters compared to robotic‐assisted primary TKA. Specifically, they compared 20 robotic UKA to TKA conversions with 20 primary robotic TKAs. The values of the OLA, LDFA, MPTA and slope angles between the two groups did not show significant differences.

In all cases enroled in this study, the acquisition of intraoperative landmarks was possible in the same way as with primary implants, so it was never necessary to abort the use of the robot during the surgical procedure.

Furthermore, no intraoperative complications were reported.

In the study published by Mancino et al., no patient required intraoperative conversion to conventional manual instrumentation for TKA, and the complication rate between patients treated with primary robotic TKA and patients undergoing revision from UKA to TKA with robotic technique did not show statistical significance [21]. In the case series reported by Lachance et al. on the conversion of patellofemoral implants with TKA, no intraoperative complications were reported [15].

The surgical times were in line with both the conventional technique and other studies on robotic‐assisted revision surgeries [22, 38]. In detail, the data reported in this study indicate an average time of 116.1 (SD 19.6) min. In the study by Mancino et al., involving 16 patients treated with robotic revision TKA for UKA implant failure compared with 35 patients treated with primary robotic TKA, a statistically significant difference in average surgical time emerged [21]. The revision group reported an average time of 127 (SD 18) min.

The data on the length of stay are lower in the studies reported in the literature, ranging from 1.3 to 2.7 days; however, they are not comparable as they are framed within healthcare systems of other countries [21, 38].

The data in the literature report up to 100% use of primary implants during the robotic revision procedure from UKA to TKA. Magruder et al. reported the use of uncemented cruciate‐retaining (CR) implants in 86.5% of cases and an additional 9.1% of cemented CR implants [20]. At the tibial level, 1.3% of cases involved the use of tibial baseplates and 9.1% involved medial tibial augments.

Yun et al. treated all patients with CS (cruciate substituting) primary implants, noting that the choice was largely based on surgeon preference rather than on indication of posterior cruciate ligament insufficiency [38]. Regarding polyethylene thickness, the average thickness in the robotic group was 10 mm, with a range from 9 to 14 mm. No statistical difference in polyethylene thickness was observed when compared to patients treated using conventional techniques (*p* = 0.07).

In the study by Mancino et al., primary PS implants were used in 82% of cases, and varus‐valgus‐constrained implants in 18% [21]. The thinnest insert, corresponding to 9 mm in this type of implant, was used in 63% of the cases.

In the study by Tuecking et al., standard bicruciate‐stabilized liners were used in 90% of the cases involving the conversion of UKA, while varus‐valgus‐constrained implants were used in 10% of the cases [35]. The thinnest liner was employed in the majority of cases within the UKA revision group (70%).

In the research published by Magruder et al. involving 44 patients who underwent robotic revision TKA following previous UKAs, the patient‐reported outcome measures at the 1‐year follow‐up showed significant improvements [20]. The average Knee Injury and Osteoarthritis Outcome Score, Joint Replacement score rose from 48.1 to 68.7 (*p* < 0.001), and the WOMAC score decreased from 25.7 to 10.6 (*p* = 0.003).

In this study, the WOMAC score at the final follow‐up was 17.8 (SD 8.7), showing a statistically significant improvement compared to before the revision (53.5 ± 21.3; *p* < 0.001).

Mancino et al. reported a ROM of 119° of knee flexion at the average final follow‐up of 21 months (ranging from 6 to 36 months), similar to the data from this study which reports 119.4° (SD 13.4) at the final follow‐up of 31.3 months (SD 12.1) [21]. A significant finding from the study by Mancino et al. is that at the final follow‐up, the primary robotic arm‐assisted TKA group showed a statistically significant improvement in the OKS compared to the revision UKA to TKA group (44.6 [SD 2.7] vs. 42.3 [SD 2.5]; *p* = 0.004). However, there was no difference in overall ROM (*p* = 0.056) or the FJS between the two treatment groups (86.1 [SD 9.6] vs. 84.1 [SD 4.9]; *p* = 0.439).

This contrasts with the findings of Sun et al., where WOMAC, KSS scores and ROM were superior in primary TKAs compared to the group undergoing UKA to TKA revisions with conventional technique [34]. The use of robotic techniques may influence this aspect, as robotic assistance can lead to better soft tissue balance, increased bone tissue preservation and consequently, greater patient well‐being.

At the final follow‐up of 31.3 ± 12.1 months, this study reported an implant survival rate of 97.14%. Similarly encouraging data are reported by Mancino et al., with a reoperation rate of 0% at the average final follow‐up of 21 months [21]. Magruder et al. also reported an overall survivorship of 93.18% at 1.8 years and an aseptic survivorship of 97.73% [20].

This study has several limitations. First of all, the sample size is small and it is a prospective single cohort study without a control group. The follow‐up period is short and a sample size was not calculated before starting

However, compared to other studies in the literature that evaluate patients treated with robotic techniques for the revision of UKA to TKA, this sample has a larger number of participants and a longer follow‐up period and each case was performed using a described and reproducible surgical technique, ensuring consistency across cases. The results have been evaluated and compared with the outcomes of previous studies on a similar population.

## CONCLUSIONS

The current results and survivorship are promising, indicating that robotic assistance can be a useful tool for revising UKA to TKA, both for the failure of the previous implant and for tricompartmental OA progression. This study highlights that in the cohort of patients undergoing robotic‐assisted conversion from UKA to TKA, the use of an imageless procedure through intraoperative bone morphing combined with alignment based on functional philosophy has proven to be safe and has resulted in excellent clinical and radiographic outcomes.

## AUTHOR CONTRIBUTIONS

Luca Andriollo and Stefano Marco Paolo Rossi designed and Luca Andriollo was responsible for the study. Luca Andriollo and Rudy Sangaletti contributed to write the manuscript and finalize the statistical section. Virgina Cinelli and Calogero Velluto were responsible for the clinical follow‐up of the patients. Stefano Marco Paolo Rossi and Francesco Benazzo supervised and revised the manuscript.

## CONFLICT OF INTEREST STATEMENT

F. B. receives royalties and has a consulting contract with Zimmer Biomet. S. M. P. R. has a consulting contract with Zimmer Biomet. The remaining authors declare no conflict of interest.

## ETHICS STATEMENT

The study was approved by the Ethical Committee of our institution (IRB approval No. NK 5022). All patients signed an informed consent for the surgical procedure and for publication of the data. This manuscript is original and not published elsewhere.

## Data Availability

Data are available upon request on a separate Data Repository.
